# Battling herbivores: the volatile story of tea

**DOI:** 10.1093/plphys/kiaf441

**Published:** 2025-09-26

**Authors:** Nilesh D Gawande

**Affiliations:** Assistant Features Editor, Plant Physiology, American Society of Plant Biologists; Department of Biotechnology, School of Sciences, Woxsen University, Hyderabad, Telangana 502345, India

Tea plants (*Camellia sinensis*) are prone to herbivore attacks, which can cause severe yield losses. Plants possess strong systemic defense mechanisms to control such infestations. First, the plant recognizes the attack and then activates a defense response, which may include the production of toxic volatile compounds to deter herbivores or the formation of volatile compounds that attract their predators. The plant volatile compound (E)-4,8-dimethyl-1,3,7-nonatriene (DMNT) plays a critical role in defense against multiple insect pests, as well as in attracting natural enemies or predator species of the pest (Arimura et al. 2009). The activation of these responses often involves various signaling pathways, including ethylene, jasmonic acid (JA), and salicylic acid (Howe and Jander 2008; Fonseca et al. 2014).

The JA pathway possesses a strongly conserved mechanism for defense against arthropod herbivores (Howe and Jander 2008). JA and its bioactive derivatives accumulate at the damage site in response to herbivore infestation, which is perceived by the jasmonate co-receptors. The JA derivative (+)-7-iso-jasmonoyl-L-isoleucine (JA-Ile) is the main active JA derivative that controls vascular plant responses to herbivores and JA-regulated processes (Fu et al. 2022).

The tea geometrid (*Ectropis obliqua*) is a lepidopteran moth species and a common insect pest that feeds on the leaves and tender buds of tea gardens in China, causing deterioration in quality as well as yield losses in tea plants.

**Figure 1.**
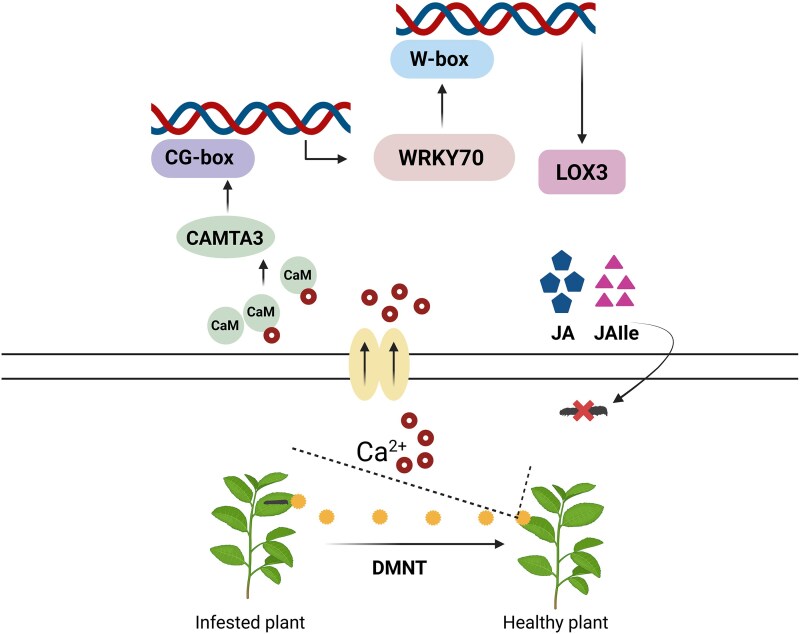
Mechanism of action of the CAMTA3–WRKY70–LOX3 module in defense against herbivory. When the tea geometrid infests tea plants (*Ectropis obliqua*), they release DMNT, which signals nearby healthy plants. This triggers an influx of Ca²⁺ into the mesophyll cells, resulting in a change in Ca²⁺ concentration. Consequently, the CAMTA3–WRKY70–LOX3 module is activated, accumulating JA and JA-Ile and conferring resistance against herbivory. The figure is created with BioRender.com, with a few modifications to the figure from Li et al. (2025).

In this issue of *Plant Physiology*, Li et al. (2025) demonstrated how the volatile compound DMNT, induced by herbivore attack, activates the JA biosynthesis pathway in tea plants (Fig. 1). In previous studies, the authors determined that in response to *E. obliqua* infestation, tea plants release herbivory-induced plant volatiles, which cause an increase in JA and JA-Ile levels in surrounding non-infested plants, alerting them to the herbivory infestation. The analysis of volatile compounds released by *E. obliqua* using gas chromatography-mass spectrometry showed that DMNT was the prominently induced compound after the herbivory attack. DMNT-exposed tea leaves showed increased levels of JA and JA-Ile and induced JA biosynthesis genes, including *CsLOX3*. In contrast, JA biosynthesis gene expression was abolished in plants treated with JA inhibitors. JA inhibitor-pretreated plants showed more larval feeding than plants exposed to DMNT and did not exhibit an anti-herbivory response to DMNT, suggesting that *E. obliqua* infestation induces DMNT, activating the JA-dependent resistance in neighboring tea plants.

Transcriptome analysis of neighboring tree plants indicated that various transcription factors and JA biosynthesis genes were induced. A strong correlation was detected between the expression of *CsLOX3*, transcription factor CsWRKY70, and another gene. Subcellular localization using eYFP showed that CsWRKY70 is localized in the nucleus. The interaction between the transcription factor CsWRKY70 and the *CsLOX3* promoter was confirmed through a yeast 1-hybrid assay and a dual luciferase assay. CsWRKY70 has a WRKY-DNA binding domain that recognizes the W-box motif, while *CsLOX3* has a W-box cis-acting element. The electronic mobility shift assay demonstrated that CsWRKY70 directly binds to the W-box element in the *CsLOX3* promoter region.

The silencing of the *CsWRKY70* gene in tea leaves using an antisense oligonucleotide significantly reduced the expression of the *CsLOX3* as well as the levels of JA and JA-Ile. In contrast, plants expressing *CsWRKY70* showed the opposite effect, with higher levels of these compounds. Feeding *E. obliqua* larvae on the tea leaves with silenced *CsWRKY70* resulted in increased leaf damage and a significant elevation in larval weights compared with the control. The exogenous application of DMNT in *CsWRKY70*-silenced plants resulted in reduced expression levels of *CsWRKY70* and *CsLOX3* along with the downregulation of the biosynthesis of JA and JA-Ile genes. This indicates that CsWRKY70 enhances resistance to *E. obliqua* through *CsLOX3* expression and DMNT-mediated JA induction.

Furthermore, tea seedlings exposed to DMNT and other herbivore-induced plant volatiles showed that short exposure to DMNT induces *CsWRKY70* and *CsLOX3*. DMNT enhances the luciferase activity of CsWRKY70, while other compounds reduce CsWRKY70 activity, indicating that DMNT specifically induces *CsWRKY70*.

During biotic stress conditions, such as plant-insect interactions, calcium (Ca²⁺) acts as a secondary messenger, and herbivory attacks induce changes in the cytosolic Ca²⁺ through various channels and signaling pathways (Malabarba et al. 2021; Kloth and Dicke 2022). Therefore, the authors measured transmembrane Ca²⁺ flux in mesophyll cells of tea leaves after exposure to DMNT. Increasing DMNT concentrations rapidly elevated Ca²⁺ influx in mesophyll cells. Pretreatment with LaCl₃, a competitive inhibitor of Ca²⁺ channels, reduced Ca²⁺ influx, indicating the involvement of Ca²⁺ channels in DMNT-induced Ca²⁺ influx. *CsCML42* and *CsCDPK1* were induced following short DMNT exposure. These results suggest that DMNT mediates signaling that triggers Ca²⁺ influx in mesophyll cells by regulating the expression of calmodulin and its associated protein kinases.

The CsWRKY70 promoter contains a CG-box cis-acting element, recognized as a specific binding site for calmodulin-binding transcription activator (CAMTA) transcription factors. Yeast 1-hybrid assays, electrophoretic mobility shift assay, and dual luciferase assays revealed that only CAMTA3 from tea plants binds to the CG-box of the CsWRKY70 promoter, indicating that CAMTA3 regulates CsWRKY70 expression. Expression analysis using qRT-PCR showed that DMNT exposure significantly induced *CAMTA3*, while silencing *CAMTA3* with antisense oligonucleotide reduced the expression of *CsWRKY70*, and *CsLOX3*, as well as decreased the accumulation of JA and JA-Ile upon DMNT exposure. These findings suggest that CAMTA3 plays a critical role in regulating *CsWRKY70* expression in response to DMNT and enhances JA and JA-Ile biosynthesis.

Overall, this study demonstrates that DMNT activates the CAMTA3-WRKY70-LOX3 module through the Ca^2+^ signaling pathway, resulting in JA and JA-Ile biosynthesis and activating herbivore resistance in tea plants. It will be interesting to further study the molecular mechanism of how herbivore-induced plant volatiles other than DMNT impart herbivore resistance in tea plants.
